# Association of recent gay-related stressful events with depressive symptoms in Chinese men who have sex with men

**DOI:** 10.1186/s12888-018-1787-7

**Published:** 2018-07-04

**Authors:** Yunyong Liu, Chao Jiang, Siyao Li, Yuan Gu, Yan Zhou, Xiaoxia An, Li Zhao, Guowei Pan

**Affiliations:** 1 Cancer Hospital of China Medical University, Liaoning Cancer Hospital & Institute, Xiaoheyan Road 44, Dadong District, Shenyang, 110042 People’s Republic of China; 2 Department of Psychiatry, Dalian Medical University, Dalian, People’s Republic of China; 3 Department of Psychiatry, Liaoning Provincial People’s Hospital, Shenyang, People’s Republic of China; 4 Shenyang Municipal Center for Disease Control and Prevention, Shenyang, People’s Republic of China; 5 Dandong Municipal Center for Disease Control and Prevention, Dandong, People’s Republic of China; 6 Benxi Municipal Center for Disease Control and Prevention, Benxi, People’s Republic of China; 7 Anshan Municipal Center for Disease Control and Prevention, Anshan, People’s Republic of China; 8 Liaoning Provincial Center for Disease Control and Prevention, Shenyang, People’s Republic of China

**Keywords:** Men who have sex with men, Gay related stressful event, Depressive symptoms, Social support

## Abstract

**Background:**

To assess the association of different gay-related stressful events (GRSEs) with depressive symptoms in Chinese men who have sex with men (MSM).

**Method:**

A total of 807 MSM were recruited using respondent-driven sampling from four cities in northeastern China. GRSEs were measured using the Gay Related Stressful Life Events Scale, and depressive symptoms were assessed using the Self-Rating Depression Scale (SDS).

**Results:**

A total of 26.0% of study participants experienced GRSEs in the past three months, and the average SDS score was lower than the previously reported national average for China. The study participants had significantly elevated risks of depression (SDS score ≥ 53) due to recent troubles with a boss (OR = 4.92, 95% CI = 1.87–12.97) or a workmate (OR = 3.68, 95% CI = 1.52–8.88), loss of a close friend (OR = 2.41, 95% CI = 1.39–4.18), argument with a close friend (OR = 2.07, 95% CI = 1.33–3.22), and being physically assaulted (OR = 2.08, 95% CI = 0.98–4.43). Arguments with family members or classmates had no significant effect on depression. Multiple logistic regression analysis showed that the number of GRSEs, a lower level of education, more advanced age, and HIV infection significantly increased the risk of depression.

**Conclusions:**

There are large differences in the associations of different types of GRSEs with depressive symptoms. Reducing the stigmatization and discrimination toward MSM in all social environments and improving the capability of MSM to cope with different types of GRSEs may improve their emotional wellbeing.

## Background

Homosexuality has been traditionally stigmatized and prejudiced in many countries, men who have sex with men (MSM) have been identified as a higher risk group for depression as compared with general populations [1–3]. Many studies reported that MSM experience high levels of gay-related stressful events (GRSEs) from multiple life domains (e.g.,family, work, school, relationship) because of their gay behaviors or orientation [4–6]. The total population of Chinese MSM has been estimated at 17.82 million [7], they are particularly vulnerable to psychiatric disorders compared with heterosexual males, the lifetime prevalence of major depression disorder and suicide attempt were about 2.2 and 5.8 times greater than that of male adults in the general population of China [8, 9]. Social support refers to the provision of psychological and material resources by people within one’s social network [10]. As an isolated and marginalized population, MSM has inadequate level of social support, which has been shown to act as a ‘stress buffer’ to mitigate depression [11, 12]. It’s estimated that 77,000 Chinese MSM have been infected with human immunodeficiency virus (HIV) [13], and many studies have demonstrated that the health concern about HIV infection has become the most prominent health risk and source of stress of MSM [14]. Some studies suggested that the experienced GRSEs, lack of social support and the high rate of HIV infection among gay men contributed to their higher prevalence of poor mental health [15–18], and researchers have suggested that these additional external stressors may directly lead to psychosocial difficulties; or indirectly via internal factors (like internalized homophobia), through a process termed minority stress [19].

Previous studies in western countries have identified that the type and intensity of GRSEs is related to the extent of psychological dysfunction [1–6, 20, 21]. However, few studies have assessed the associations between different types of GRSEs and depressive symptoms in Chinese MSM. A better understanding of characteristics of the relationships between various types GRSEs and emotional distress in these men has clear implications for reducing their stress and improving their mental health.

The present study aimed to assess the following hypotheses regarding Chinese MSM: (1) recent GRSEs correlate with depressive symptoms; (2) different types of GRSEs have distinct effects on depressive symptoms; (3) concern about HIV infection and lack of social support increases the risk for depressive symptoms.

## Methods

We have described the selection of MSM of the study previously [8]. In brief, 807 MSM were recruited from four cities of Liaoning Province using a standardized respondent-driven sampling (RDS) procedure. Respondents were included if they (1) had oral or anal sexual relations with another man during the previous 12 months, (2) 18 to 65 years-old, (3) agreed to complete a questionnaire, (4) provided blood samples for testing. The trained interviewers used a structured questionnaire to interview all subjects, without asking names and other personal information, including personal identification numbers.

The study was conducted in accordance with the Declaration of Helsinki. Verbal consents were obtained from all subjects prior to interview. The ethics committee of Liaoning Provincial Center for Disease Control and Prevention (LNCDCP) approved the study.

### Depressive symptoms

Depression symptoms were measured with the 20 items Self-Rating Depression Scale (SDS) [22]. Each item was scored from 1 to 4 (never or occasionally, sometimes, often, and most of the time), and multiplied by 1.25 to obtain a total score of SDS ranging from 25 to 100. The subjects with an SDS score ≥ 53 were classified into the depression group, the internal consistency (Cronbach’s alpha) of the SDS was 0.92 [23].

### Gay-related stressful life events

GRSEs were measured with the 12 items Gay Related Stressful Life Events Scale [24], which measures the occurrence of incidents related to sexual orientation over the past three months, including the arguments with friends and relatives; conflicts in the home, society and workplace; and loss of friends and victimization. The total score was calculated by summing the number of items checked, and a higher score indicates greater stress.

### Social support

Social support was measured by the 10 items Social Support Rating Scale (SSRS) [25], which measure subjective support (4 items), objective support (3 items), and support-seeking behavior (3 items). The scores of 3 dimensions of SSRS were added to generate a total score of 0 to 50, and a higher score indicates stronger social support. The SSRS has good reliability and validity [25], the internal consistency (Cronbach’s alpha) was 0.89–0.94 and the test-retest reliability is 0.92.

### HIV testing

HIV was tested with the Chinese national standard protocols and laboratory methods [26]. An enzyme-linked immunosorbent assay (ELISA) method was used to conduct the initial screening, and positive results were confirmed by a western blotting. We defined a result as positive only if both tests were positive. All laboratory tests were performed in the AIDS labs of LNCDCP.

### Statistical methods

We used SPSS, version 17.0 to conduct the data analysis. We used the analysis of variance (ANOVA) to determine the significance of relationships of demographic characteristics, types of GRSEs, and SDS score. We used a linear trend test to examine differences in mean changed of SDS scores by the number of GRSEs. We conducted bivariate logistic regression analyses of the presence/absence of depression (SDS ≥ 53) for calculation of crude odds ratios (ORs) and 95% confidence intervals (CIs) to examine the associations with each type of GRSEs with adjustment for age. We conducted stepwise multivariate logistic regression analyses to calculate the adjusted ORs.

## Results

Table 1 shows the basic characteristics of the 807 recruited MSM. A total of 71.3% were younger than 30 years-old, 13.4% were married to females, 11.4% lived with male partners, 40.3% were bisexual, and 47.2% were gay, and 3.47% were HIV-positive. The average SDS score was 47.29 ± 11.39, and the overall prevalence of depression (SDS ≥ 53) was 33.09%.

**Table 1:** Comparison of the levels of SDS scores and risks of depression (SDS score ⩾ 53) by demographic characteristics of Chinese men who have sex with men (*n* = 807)

	N	%	SDS score			Depression (SDS score ⩾ 53)		
			Mean	SD	*p* value	OR	95% CI	
Age, years								
18–29	575	71.30	46.24	11.24	*< 0.001*	1.00		
30–39	133	16.50	48.29	11.31		1.28	0.86	1.91
40–64	99	12.30	52.01	11.12		*2.79*	*1.81*	*4.31*
p for trend			< 0.001					
Education, years								
< 10	255	31.60	49.34	11.05	*< 0.001*	1.00		
10–12	322	39.90	47.74	11.61		0.80	0.57	1.12
⩾13	230	28.50	44.37	10.88		*0.45*	*0.30*	*0.67*
p for trend			< 0.001					
Income								
Low	305	37.79	47.16	11.84	0.046	1.00		
Middle	280	34.70	48.48	10.79		1.07	0.76	1.51
High	222	27.51	45.96	11.37		0.81	0.56	1.18
p for trend			0.32					
Relationship status								
Single	571	70.76	46.34	11.26	*0.003*	1.00		
Married with a female	108	13.38	49.97	10.89		*1.89*	*1.24*	*2.88*
Cohabitation with a male	92	11.40	49.20	11.63		1.45	0.92	2.29
Divorced	36	4.46	49.34	12.47		1.58	0.78	3.17
Sexual identity								
Heterosexual/Unconfirmed	101	12.52	45.85	11.30	0.397	1.00		
Gay	381	47.21	47.55	11.30		1.26	0.78	2.03
Bisexual	325	40.27	47.42	11.51		1.27	0.78	2.07
HIV positive								
No	779	96.53	47.07	11.30		1.00		
Yes	28	3.47	53.35	12.32	*0.004*	*3.83*	*1.74*	*8.42*
SSRS level								
Low	276	34.20	43.99	10.99	*< 0.001*	1.00		
Middle	264	32.71	46.92	10.82		*0.49*	*0.35*	*0.71*
High	267	33.09	51.06	11.25		*0.57*	*0.48*	*0.69*
p for trend			< 0.001					

OR, odds ratio adjusted for age; HIV, human immunodeficiency virus; SDS, self-rating depression score;⩾53, cut-off value for SDS score; SSRS, social support rating score; Italic OR: *p* < 0.05

The levels of SDS score had a significant linear increase with age (*p* < 0.001), and significant linear decrease with education (*p* < 0.001) and social support (*p* < 0.001). The mean SDS score was significantly greater in those who experienced a GRSE (*p* < 0.001) and in those infected with HIV (*p* = 0.004). The risks of depression (SDS score ≥ 53) were significantly elevated in those who were aged over 40 (OR = 2.79, 95% CI = 1.81–4.31), married with a female (OR = 1.89, 95% CI = 1.24–2.88), experienced a GRSE (OR = 1.68, 95% CI = 1.22–2.33), and were infected with HIV (OR = 3.83, 95% CI = 1.74–8.42), but significantly reduced in those with highest education years (OR = 0.45, 95% CI = 0.30–0.67).

Table 2 shows that 26.0% of the subjects experienced at least 1 GRSE during the previous 3 months, and 9.8% had 2 or more such events. The 5 most common types of events were arguments with close friends (11.3%), losing a close friend (6.8%), arguments with parents (6.4%), arguments between parents (3.6%), and physical assault (3.5%). The SDS score was significantly greater in those who experienced any GRSE, an argument with a close friend, the loss of a close friend, trouble with a workmate, trouble with a boss or supervisor, and physical assault. The highest ORs for depressive symptoms were in those who experienced trouble with a boss or supervisor (OR = 4.92, 95% CI = 1.87–12.97), trouble with a workmate (OR = 3.68, 95% CI = 1.52–8.88), loss of a close friend (OR = 2.41, 95% CI = 1.39–4.18), and argument with a close friend (OR = 2.07, 95% CI = 1.33–3.22). There was also a significant linear relationships of the number of GRSEs with SDS score (*p* < 0.01).

**Table 2:** Comparison of the levels of SDS scores and risks of depression (SDS score ⩾ 53) among Chinese men who have sex with men (*n* = 807)

GRSEs	N	%	SDS score					Depression (SDS score ⩾ 53)		
			Yes		No		*p* value	OR	95% CI	
			Mean	SD	Mean	SD				
Arguments with a close friend about your SSB	91	11.28	*50.73*	*11.92*	*46.85*	*11.25*	*< 0.01*	*2.07*	*1.33*	*3.22*
Losing a close friend because of your SSB	55	6.82	*52.16*	*9.86*	*46.93*	*11.41*	*< 0.01*	*2.41*	*1.39*	*4.18*
Arguments with parents about your SSB	52	6.44	49.88	10.77	47.11	11.41	0.09	1.40	0.79	2.49
Arguments between your parents about your SSB	29	3.59	48.10	11.28	47.26	11.39	0.69	0.91	0.41	2.02
Being physically assaulted in a “gay-bashing” incident	28	3.47	*52.41*	*10.32*	*47.10*	*11.38*	*0.02*	2.08	0.98	4.43
Trouble with a workmate or workmates over your SSB	22	2.73	*53.07*	*12.04*	*47.12*	*11.33*	*0.02*	*3.68*	*1.52*	*8.88*
Trouble with your boss or supervisor about your SSB	20	2.48	*56.06*	*9.78*	*47.06*	*11.34*	*< 0.01*	*4.92*	*1.87*	*12.97*
Arguments with other family members about your SSB	17	2.11	48.90	13.22	48.90	13.22	0.56	1.82	0.70	4.78
Trouble with a brother or sister about your SSB	14	1.73	48.75	14.01	47.26	11.34	0.63	2.05	0.71	5.91
Trouble with classmates over your SSB	9	1.12	48.47	14.84	47.27	11.35	0.75	0.57	0.12	2.79
Getting in trouble with the police because of your SSB	8	0.99	50.47	12.75	47.25	11.37	0.43	2.04	0.51	8.21
Trouble with your teachers over your SSB	3	0.37	39.58	5.05	47.31	11.39	0.24	Blank^a^		
Any trouble above	210	26.02	49.64	11.39	46.46	11.28	*< 0.01*	*1.68*	*1.22*	*2.33*
Number of events										
0	597	73.98	46.50 ± 11.28				*< 0.01*	1.00		
1	131	16.23	47.77 ± 11.46					1.18	0.79	1.77
≥2	79	9.79	52.74 ± 10.62					*2.94*	*1.82*	*4.73*
P for trend			*< 0.01*							

SDS, self-rating depression score; ⩾53, cut-off value for SDS score; OR, odds ratio adjusted for age; SSB, same-sex sexual behavior

^a^OR could not be calculated because none experience such a GRSE in the group with SDS score < 53; Italic OR: *p* < 0.05

Table 3 shows the results of multiple logistic regression analysis of factors associated with depression (SDS ≧ 53). These results indicate that age of 40 years or more (AOR = 2.58, 95%CI = 1.64–4.07), HIV infection (AOR = 3.48, 95% CI = 1.49–8.10), and the recent experience of 2 or more GRSE (AOR = 3.19, 95% CI = 1.93–5.26) significantly increased the risk of depression. However, 13 or more years of education (AOR = 0.57, 95% CI = 0.40–0.83) and greater social support (mid-level SSRS score: AOR = 0.54, 95% CI = 0.37–0.79; high-level SSRS score: AOR = 0.59, 95% CI = 0.49–0.72) protected against depression.

**Table 3:** Multiple regression analysis of factors associated with depression (SDS score ≥ 53) among Chinese men who have sex with men (*n* = 807)

	β coefficient	Wald statistics	AOR	95% CI		*p* value
Education: ≥13 years	−0.56	8.64	0.57	0.40	0.83	0.003
Age: ≥40 years	0.95	16.69	2.58	1.64	4.07	< 0.001
HIV positive	1.25	8.36	3.48	1.49	8.10	0.004
SSRS level						
Middle	−0.61	10.48	0.54	0.37	0.79	0.001
Highest	−0.52	27.66	0.59	0.49	0.72	< 0.001
Number of GRSEs						
1	0.19	0.76	1.21	0.79	1.85	0.383
⩾2	1.16	20.46	3.19	1.93	5.26	< 0.001

*AOR* Adjusted odds ratio, *SSRS* social support rating score

## Discussion

We used a standardized respondent-driven sampling (RDS) procedure to sample 807 MSM from four cities in Liaoning Province, China. Similar to previous findings that Chinese MSM had higher level of depressive symptoms, but lower social support than their heterosexual peers [27–31]. The rate of depression (SDS ≧ 53) in our subjects (33.09%) is similar to that reported for MSM from Foshan City, China (34.8%) [27], but lower than in several other reports of Chinese MSM (45.5–67.0%) [28–31]. These discrepancies might be due to differences in culture, social-economic status, age distribution, and rate of HIV infection [27–31]. They could also be due to differences in the cutoff value used to define depression, or differences in sampling methods.

More than one-fourth of our subjects experienced GRSEs in their daily lives during the past 3 months, confirming the high prevalence of antigay discrimination and prejudice encountered by MSM in China and elsewhere [2, 4–6]. Although arguments with family members and teachers or classmates regarding same-sex sexual behavior (SSB) are common GRSEs, they had no significant effects on SDS score (Table 2). This may be because MSM have learned to tolerate conflicts in the family or school due to their frequent occurrence over long periods. On the other hand, recent troubles with a boss or a workmate had the greatest effects on depressive symptoms, followed by loss of a close friend, argument with a close friend, and being physically assaulted. These results suggest that GRSEs from non-family members are the major stressors for Chinese MSM, and support our hypothesis that the types, places, and persons involved in GRSEs have significantly different effects on the levels of emotional distress [1–3, 20, 21]. These results also support the idea that the occurrence of life events is less important than how they are perceived, and that it is therefore important to measure an individual’s perceptions of different life events and the ensuing stress [32].

Our finding that depressive symptoms were strongly associated with troubles involving a boss or workmate suggests that MSM find managing GRSEs in the workplace more difficult than in other aspects of life. Thus, even if these events are episodic, they are more difficult to manage than GRSEs within the family or school, and again highlight the significance of the setting and person involved in GRSEs [33]. The strong association of depressive symptoms with losing a close friend or arguing with a close friend indicates that a close personal network is important social capital for Chinese MSM, presumably because it helps them maintain their mental stability and health. Disruption of an individual’s personal network is not only an acute stressor in itself, but may also reduce an individual’s ability to cope with other GRSEs because it can influence cognition, emotions, behaviors, and biological responses [34]. Our finding of significant associations between SDS score and the number of GRSEs (*r* = 0.164, *p* < 0.001) and SSES score (*r* = − 0.218, p < 0.001), and the significantly elevated risk of depression in those older than 40 years and with less education, confirm the critical role of socioeconomic factors in causing mental health problems in MSM [16–18, 35].

## Conclusion

The results of the study confirm our hypothesis that the greater depressive symptoms of Chinese MSM may be accounted for, at least in part, by exposure to GRSEs and a lack of social support due to their status as a sexual minority [1–6]. This also provides further evidence that establishing and maintaining social cohesion is very important for maintaining the mental health of Chinese MSM [17, 18]. HIV infection had the strongest association with depressive symptoms in the multiple regression analysis, the prevalence of HIV infection was 2.43, 4.51 and 8.08%, respectively, for those aged < 30, 30–39 and ≥ 40 years old, which may partly related to the significantly increasing levels of SDS with age. This confirms that events related to AIDS have strong correlations with emotional distress in MSM [14, 32]. Alternatively, it is possible that depressive symptoms disrupted self-regulatory processes in MSM, so that depressed individuals may be more likely to engage in unprotected sex in an effort to cope with or alleviate negative mental states [14, 31, 36–39]. We believe that both mechanisms may be involved.

### Limitations

There are several limitations of this study. First, the temporal misalignment between GRSEs (in the past three months), depressive symptoms (in the past week), and social support (not specified) may have biased the reported associations. Second, the cross-sectional design of this study made it impossible to determine causal relationships between GRSEs and depressive symptoms. Third, gay-related stress is multidimensional, so our findings may be limited by use of a 12-item questionnaire that may not have assessed some important GRSEs. Fourth, the assessment of GRSEs, depressive symptoms, and social support may have been biased because these were all self-reported. Finally, the 807 MSM were selected from four cities of northeastern China, so our findings may not be generalizable to other regions of China.

Despite these limitations, our findings provide clear evidence that GRSEs are significantly associated with greater depressive symptoms in Chinese MSM, and that different types of GRSEs have different effects. We believe that the problems of GRSEs, HIV infection, lower social support, and lower socioeconomic status co-occur at high rates and interact synergistically, leading to the higher levels of depression in Chinese MSM. More efforts are needed to increase the tolerance and reduce the stigmatization and discrimination toward MSM in all social environments within China, to improve the capability of MSM to establish and maintain strong personal social support networks, and to teach MSM to better manage different types of GRESs.

## Abbreviations

CIs: Confidence intervals
GRSEs: Gay related stressful events
LNCDCP: Liaoning Provincial Center for Disease Control and Prevention
MSM: Men who have sex with men
NLEs: Negative life events
ORs: Odd ratios
RDS: Standardized respondent-driven sampling
SDS: Self-rating depression scale
SSB: Same-sex sexual behavior
SSRS: Social support rating scale
